# Evolutionary Signatures amongst Disease Genes Permit Novel Methods for Gene Prioritization and Construction of Informative Gene-Based Networks

**DOI:** 10.1371/journal.pgen.1004967

**Published:** 2015-02-13

**Authors:** Nolan Priedigkeit, Nicholas Wolfe, Nathan L. Clark

**Affiliations:** 1 Medical Scientist Training Program, University of Pittsburgh School of Medicine, Pittsburgh, Pennsylvania, United States of America; 2 Department of Pharmacology and Chemical Biology, University of Pittsburgh, Pittsburgh, Pennsylvania, United States of America; 3 Department of Computational and Systems Biology, University of Pittsburgh, Pittsburgh, Pennsylvania, United States of America; University of Washington, UNITED STATES

## Abstract

Genes involved in the same function tend to have similar evolutionary histories, in that their rates of evolution covary over time. This coevolutionary signature, termed Evolutionary Rate Covariation (ERC), is calculated using only gene sequences from a set of closely related species and has demonstrated potential as a computational tool for inferring functional relationships between genes. To further define applications of ERC, we first established that roughly 55% of genetic diseases posses an ERC signature between their contributing genes. At a false discovery rate of 5% we report 40 such diseases including cancers, developmental disorders and mitochondrial diseases. Given these coevolutionary signatures between disease genes, we then assessed ERC's ability to prioritize known disease genes out of a list of unrelated candidates. We found that in the presence of an ERC signature, the true disease gene is effectively prioritized to the top 6% of candidates on average. We then apply this strategy to a melanoma-associated region on chromosome 1 and identify *MCL1* as a potential causative gene. Furthermore, to gain global insight into disease mechanisms, we used ERC to predict molecular connections between 310 nominally distinct diseases. The resulting “disease map” network associates several diseases with related pathogenic mechanisms and unveils many novel relationships between clinically distinct diseases, such as between Hirschsprung's disease and melanoma. Taken together, these results demonstrate the utility of molecular evolution as a gene discovery platform and show that evolutionary signatures can be used to build informative gene-based networks.

## Author Summary

Molecular evolution has informed our understanding of gene function; however, classical methods have largely been static in their implementation, focusing on single genes. Here, we present and prove the utility of a dynamic, network-based understanding of molecular evolution to infer relationships between genes associated with human diseases. We have shown previously that groups of genes within functional niches tend to share similar evolutionary histories. Exploiting the availability of whole genomes from multiple species, these histories can be numerically scored and dynamically compared to one another using a sequence-based signature termed Evolutionary Rate Covariation (ERC). To explore potential applications, we characterized ERC amongst disease genes and found that many diseases contain significant ERC signatures between their contributing genes. We show that ERC can also prioritize “true” disease genes amongst unrelated gene candidates. Lastly, these signatures can serve as a foundation for creating instructive gene-based networks, unveiling novel relationships between diseases thought to be clinically distinct. Our hope is that this study will add to the increasing evidence that advancing our understanding of molecular evolution can be a crucial asset in large-scale gene discovery pursuits (Link to our webserver that provides intuitive ERC analysis tools: http://csb.pitt.edu/erc_analysis/).

## Introduction

Advances in sequencing technologies and collaborative, large-scale—omics and genome-wide association projects are providing investigators with overwhelming lists of candidate disease gene associations. In the past decade, nearly 2,000 genomic regions have been associated with over 300 complex traits, and open efforts such as The Cancer Genome Atlas have produced petabytes of genetic data to sift through [1,2]. To more effectively decipher and prove candidate genes' roles in disease processes, computational tools have been created to both prioritize and place candidate genes into some functional context for more effective experimental validation. As these candidate genes are validated and more genes become linked with functional processes, there is also an increased ability to generate multivariable genetic networks based on these observations [3,4]. Here, we show a first-of-its-kind approach to prioritize candidate disease genes and build instructive gene-based networks based on a signature of molecular co-evolution.

Proteins do not exert their function in isolation, but rather exist within intricate networks of molecular relationships that can be revealed through high-throughput analyses of protein-protein interactions, tissue-specific expressivity and shared regulatory elements to name a few. The influx of data from these experiments has been utilized to build informative tools that aggregate and interpret these observations to place input proteins into predicted functionally related pathways [5–8]. Among many other uses, these tools have served as a catalyst for gene discovery, successfully giving functional relevance to disease gene candidates from sequencing studies and helping to validate and enhance mechanistic conclusions from high-output biological screens [9,10]. The primary methods used to create these networks rely on sophisticated algorithms that weigh certain biological features based on the query genes and sometimes user-dictated parameters. These parameters include Gene Ontology (GO) terms, genomic and proteomic study results (yeast two-hybrid, ChIP-seq, physical interactome datasets, protein structure comparisons, subcellular localization, tissue specific expressivity, etc.) and even literature mining techniques such as co-occurrence in PubMed abstracts [11].

In addition to giving functional insight to query genes, similar methods have been utilized to prioritize a list of candidate genes for further downstream study. These tools typically implement “guilt by association” strategies in which a user will have a pair of gene lists–one set of genes known in the literature to be involved in a particular pathway/disease of interest (referred to as a “training set”) and another list of candidate genes that the researcher has identified as possibly being related to the process in question. Generally, these two lists are entered into an online resource and then the candidate genes are ranked based on their relationships to the training set genes using similar databases and algorithms discussed previously [12]. Gene prioritization techniques have been effectively used in accelerating transitions from large datasets to solid biological insight [13–17].

As more data is acquired and as these tools continue to become more sophisticated and more widely used, the number of disease gene associations are increasing rapidly, mirrored by the exponential growth of entries in the Online Mendelian Inheritance in Man (OMIM) Database in the past decade [18]. This permits innovative strategies to not only focus on relationships at the molecular level, but to also implement a more expansive approach and aggregate these relationships to generate novel links between diseases and disease classes. Groups of diseases that are similar, or perhaps diseases thought to be distinct entities, may share pathogenic mechanisms between them that can be uncovered by multiscale, computational approaches [19–22]. These disease-disease relationships may lend themselves to clinically impactful drug repositioning possibilities [23,24].

Another field that has benefited greatly from this revolution in data acquisition is molecular evolution. A large number of sequenced genomes from closely related species now allows comparative and evolutionary methods to be applied across the genome. One such method, evolutionary rate covariation (ERC), infers interactions between genes using only their branch-specific rates of sequence evolution in a collection of species [25,26]. Namely, genes with rates that statistically covary tend to participate in common functions or pathways. This statistical covariation results mainly from discrete pathways responding to evolutionary pressures as a single unit, thereby causing the evolutionary rates amongst genes in the pathway to fluctuate in tandem. This evolutionary signature of co-functionality, ERC, is measured as the correlation coefficient of gene-specific branch rates between a pair of genes, for which higher values approaching 1 indicate higher rate covariation. ERC has been demonstrated between functionally related genes in mammals, *Drosophila*, fungi, and prokaryotes [1,2,26–29]. In addition, statistically significant ERC signatures are found for functionally related genes within diverse functional pathways including meiosis and piRNA metabolism [28], fertilization [30], nuclear transport [29], and more than 60% of annotated protein complexes [26]. Given the ubiquity of ERC signatures, they have even been used to discover novel genes in established genetic pathways, such as in reproductive interactions between female and male *Drosophila* [31].

Here, we introduce ERC signatures to study the genetic basis of human disease, showing that molecular evolution can serve as an innovative and complementary method for gene prioritization, functional annotation, and disease network generation. We show that, in several cases, genes associated with a particular disease show significantly elevated ERC values between them. Furthermore, ERC identifies target disease genes amongst many unrelated candidate genes based solely on shared ERC values between the candidates and a training set of known disease genes. Lastly, we demonstrate via a gene-based network approach that ERC values are elevated between diseases that share related pathogenic mechanisms and that co-evolutionary signatures can unearth novel relationships between diseases thought to be distinct.

## Results

### ERC signatures are broadly elevated between genes contributing to human diseases

**Figure 1:**
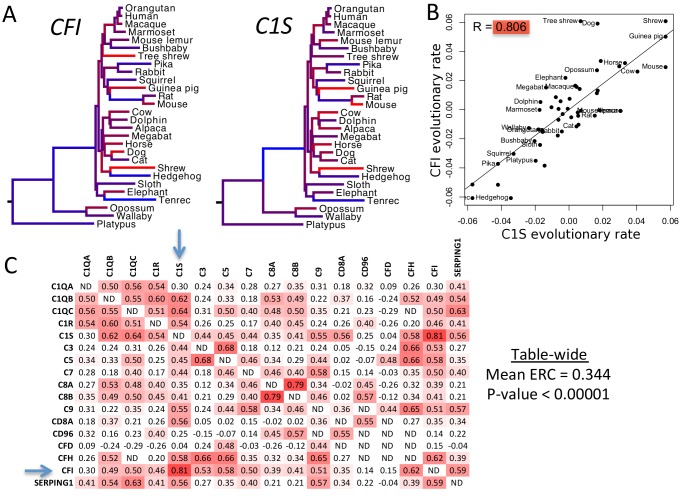
ERC values between complement deficiency genes. A) Complement genes *C1S* and *CFI* show variation in their evolutionary rates between branches of the mammalian phylogeny. Branches are color-coded according to rate. (Red is for rapid evolution, blue for slow, and intermediate shades for rates in between.) Tree topology and distances between species are the same for each gene. B) The same evolutionary rates for *C1S* and *CFI* are plotted against each other. Their correlation is apparent here in the best-fit line and correlation coefficient of 0.806. C) This matrix contains all pairwise ERC values between the OMIM genes for complement deficiency. Cells are shaded red according to the intensity of their departure from the null expectation. Blue arrows indicate the genes *C1S* and *CFI*. It is notable that most values are positive, whereas a random collection of genes would contain equal proportions of positive and negative values. There are also many clusters of functionally related complement proteins that contain very strong signals of ERC. The C1-related proteins in the upper left corner are a prime example of such an ERC hotspot.

To determine the strength of ERC signatures between disease genes we interrogated a set of 310 Disease Gene Groupings (DGG), each containing at least 3 genes known to be associated with an OMIM-annotated disease. We then examined the ERC values between each pair of constituent genes in each DGG, while testing for statistically significant elevations in ERC as a group. We first provide an example for a single DGG, complement deficiency (Fig. 1), and then continue with analysis of all DGGs. We measured evolutionary rates for complement deficiency genes *C1S* and *CFI* along all branches in a phylogeny of mammalian species. Their rates varied greatly between branches, but their patterns of variation were remarkably similar (Fig. 1A). We quantify this similarity with the Evolutionary Rate Covariation (ERC) metric, which is calculated as the correlation coefficient of their rates. Hence, the ERC value between *C1S* and *CFI* is 0.81 (Fig. 1B). All gene pairs within complement deficiency were compared in this way (Fig. 1C). Notably, the overwhelming majority of complement deficiency ERC values are positive (88%), whereas random gene sets of the same size yield positive correlation coefficients at a much lower rate (mean = 59%, maximum of 1000 nulls = 73%). Second, gene pairs with very high ERC values were found for those whose protein products form functional complexes, such as those encoding the C1 complement subcomponent: *C1Q*, *C1R*, and *C1S* (Fig. 1C, upper-left corner). An even higher ERC value was observed between members of the C8 component, *C8A* and *C8B* (ERC = 0.79). Overall, the mean ERC between all complement deficiency genes was 0.344, which yielded a highly significant p-value (permutation P < 0.00001) (Table 1).

**Table 1:** Diseases with significant ERC at a 5% false discovery rate.

Mean ERC	P-value	Q-value	N_genes_	Disease
0.870	< 0.00001	0.00002	3	Monilethrix
0.655	< 0.00001	0.00002	5	Spherocytosis
0.550	< 0.00001	0.00002	4	Cranioectodermal dysplasia
0.344	< 0.00001	0.00002	17	Complement deficiency
0.210	< 0.00001	0.00002	15	Fanconi anemia
0.188	< 0.00001	0.00002	14	Thrombophilia
0.183	< 0.00001	0.00002	16	Ichthyosis
0.130	< 0.00001	0.00002	24	Cataracts related genes
0.109	0.00002	0.00031	26	Mitochondrial complex deficiency
0.103	0.00024	0.00289	23	Immunodeficiency disorders
0.297	0.00024	0.00289	6	Hemolytic uremic syndrome
0.529	0.00039	0.00406	3	Bronchiectasis
0.273	0.00054	0.00496	6	Diamond-Blackfan anemia
0.507	0.00059	0.00522	3	Cornelia de Lange syndrome
0.503	0.00065	0.00550	3	Elliptocytosis
0.498	0.00070	0.00571	3	Hyperglycinuria
0.498	0.00070	0.00571	3	Iminoglycinuria
0.265	0.00077	0.00599	6	Homocysteine related disorders
0.050	0.00089	0.00656	65	Deafness
0.374	0.00104	0.00721	4	Thalassemia
0.048	0.00114	0.00760	69	Mental retardation
0.114	0.00130	0.00827	16	Leigh syndrome
0.451	0.00163	0.00961	3	Dysfibrinogenemia
0.174	0.00171	0.00991	9	Arrhythmogenic right ventricular dysplasia
0.134	0.00187	0.01047	12	Epidermolysis bullosa
0.145	0.00292	0.01494	10	Usher syndrome
0.160	0.00313	0.01574	9	Melanoma
0.143	0.00320	0.01600	10	Systemic lupus erythematosus
0.117	0.00511	0.02255	12	Ciliary dyskinesia
0.205	0.00531	0.02315	6	Pseudohypoaldosteronism
0.243	0.00561	0.02402	5	Aicardi-Goutieres syndrome
0.159	0.00565	0.02413	8	Muscular dystrophy-dystroglycanopathy
0.297	0.00576	0.02444	4	Paragangliomas
0.288	0.00711	0.02899	4	Asphyxiating thoracic dystrophy
0.124	0.00840	0.03302	10	Renal cell carcinoma
0.120	0.01023	0.03827	10	Malaria Susceptibility/Resistance
0.218	0.01093	0.04014	5	Thyroid dyshormonogenesis
0.265	0.01114	0.04069	4	Pituitary hormone deficiency
0.345	0.01195	0.04276	3	Maple syrup urine disease
0.075	0.01358	0.04666	18	Blood group genes

**Figure 2:**
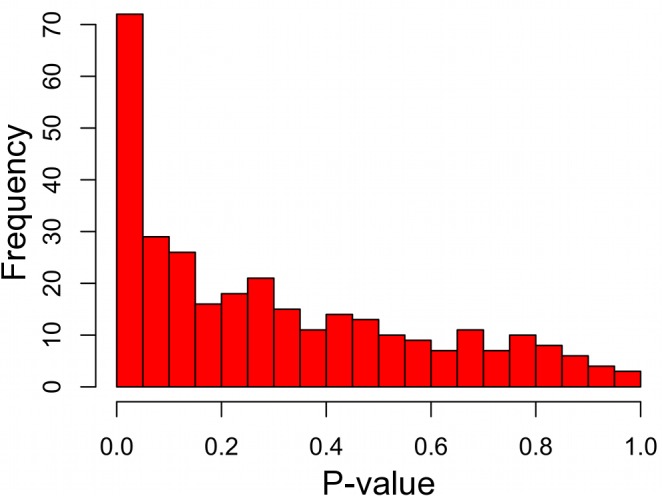
Disease gene groupings P-value distribution. P-values represent the significance of elevated mean ERC within a particular disease. There is a notable excess of low p-values, indicating a large number of diseases with an ERC signature between their genes. False discovery rate analyses show that approximately 55% of disease states interrogated have significantly elevated ERC values.

When similarly considering all 310 disease states through this analysis, 255 (82%) had positive mean ERC values, indicating a shift toward rate covariation between genes in a common disease. In contrast, random gene sets size-matched to the DGGs had positive mean values in only 59% of cases on average. The maximum observed proportion of mean positive ERCs in 1000 random sets was 69%, which is far lower than that observed for the true DGGs (82%). Moreover, there was a strong enrichment of low p-values with 73 DGGs below a nominal p-value of 0.05—a 4.7-fold excess (Fig. 2). After correction for multiple testing, 40 DGGs were found to have elevated ERC values at a false discovery rate of 5% (Table 1) [32]. From the false discovery rate analysis we also estimated that 55% of the 310 DGGs contain elevated ERC values (proportion without ERC elevation, η_0_ = 45%). Those diseases with the strongest ERC signatures included cancers, autoimmune conditions, blood cell diseases, and developmental disorders among others (Table 1). Overall, the observed significant cases indicate that pathologically related genes tend to have more positive ERC values, likely due to their analogous functions in the cell.

### ERC effectively prioritizes candidate genes for diseases with co-evolutionary signatures

We sought to assess the power of ERC co-evolutionary signatures as a gene prioritization method. Using the 310 DGGs, we asked whether a known disease gene (a “target gene” within an OMIM DGG) was effectively prioritized among a set of chromosomal neighbors using an ERC “guilt by association” approach. More specifically, candidate genes were prioritized by their ERC values with a training set of genes known to influence that disease (the remaining OMIM DGG members). Candidates with higher ERC values were more highly prioritized. To demonstrate one case, the gene *DSC2*, which contributes to arrhythmogenic right ventricular dysplasia, was tested as a “target” and its chromosomal neighbors within a 1 Mb window were treated as additional candidates. The remaining 7 genes in that disease were designated as the training set. ERC values between the training set and the target *DSC2* were 0.16 on average, which placed it in position 1 out of 31 total candidates (the 96^th^ percentile). This case was a successful prioritization. To produce a full statistical characterization of this strategy, the same procedure was repeated for all 2,416 OMIM disease genes in all 310 DGGs in our dataset, with a single training set gene being dropped from the training set and defined as the target gene iteratively. Of the 2,416 ERC prioritization tests, the 1 MB window surrounding the target gene contained a mean of 81 genes, a median of 62 genes (lower quartile = 40, upper quartile = 102) and had a range of 4 to 274 genes.

**Figure 3:**
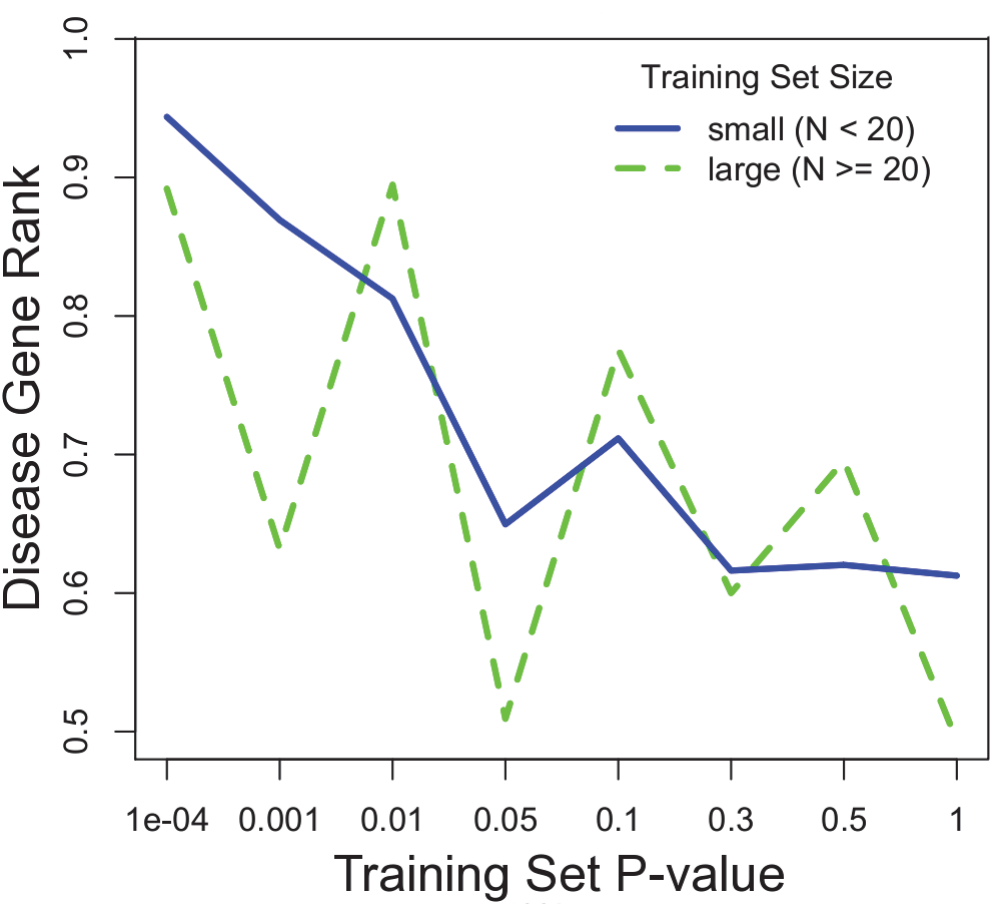
ERC disease gene prioritization. The prioritization of the true disease gene relative to its chromosomal neighbors improves with a stronger ERC signal within the training set. A low p-value (x-axis) indicates strong ERC within a training set. Prioritization (y-axis) is presented as the proportion of candidate genes scoring lower than the true disease gene, i.e. higher represents better prioritization. The blue series is for diseases with training sets with 20 or fewer genes, representing the majority (70%) of OMIM diseases interrogated. The dotted green line is for those diseases with larger training sets.

On average, ERC gene prioritization placed the target gene in the 64^th^ percentile of all candidate genes. However, the success of prioritization depended strongly on the strength of ERC within the training set (Fig. 3). When training set genes showed a significant ERC signature amongst themselves, the target gene was prioritized to a much higher position among candidates. Training sets with very strong ERC (p-value < 10^–4^) placed the target gene in the 94^th^ percentile on average (median), and training sets with ERC p-values between 10^–4^ and 10^–3^ prioritized the target gene to the 87^th^ percentile (Fig. 3). Because the strength of ERC in a training set can be determined before performing prioritization, it is a strong and practical indicator of confidence in ERC-based gene prioritization. In our scan of OMIM DGGs, small training sets (N ≤ 20 genes) prioritized target genes better than large training sets (N > 20 genes). Although large sets demonstrated a similar relationship between training set p-value and prioritization percentile, the relationship was relatively noisy. This difference was likely due to the smaller number of DGGs in this category, which resulted in higher variance in estimates of disease gene rank.

We also asked if ERC could prioritize candidate genes scattered throughout the genome instead of from a single chromosomal region. Such cases would be encountered if candidates were drawn from whole-exome sequencing data for example. ERC successfully prioritized these candidate lists as well, and almost identically to the chromosomal regions (S1 Fig.). This prioritization also demonstrated a dependency on training set p-value as observed for chromosomal regions. While low training p-values (p < 0.0001) placed the true disease gene in the 94^th^ percentile on average (median), that ranking decreased with increasing training set p-value. Overall, these tests demonstrate that ERC can be used to prioritize candidate genes from a chromosomal region or throughout the genome, especially if that disease has an ERC signature between its known genes, i.e. the training set. In the next section, we demonstrate an example application of this approach.

### ERC infers *MCL1* as a prime candidate gene within a melanoma-associated region

To demonstrate ERC gene prioritization, we prioritized candidate genes from a melanoma-associated region. Melanoma was chosen because its 9 reported causative genes have a strong ERC signature (mean ERC = 0.16, p-value = 0.00313) (Table 1), thereby providing strong predictive power as demonstrated in the previous section. A recent genome-wide study by MacGregor *et al*. found an association between melanoma susceptibility and a 430 kb region of chromosome 1q21.3 [33]. Because the region contains 10 protein-coding genes it is not clear which is causative. We prioritized these 10 candidate genes using their mean ERC signature with the 9 known melanoma genes (Table 2). One gene, myeloid cell leukemia 1 (*MCL1*), was prioritized well above the other candidates with a mean ERC of 0.173; the next highest candidate was at 0.037. The mean ERC for *MCL1* was even greater than that between the genes in the training set (0.160). Fittingly, *MCL1* encodes a protein that regulates apoptosis and cellular differentiation, and hence is a strong candidate for involvement in melanoma susceptibility [34].

**Table 2:** ERC gene prioritization for melanoma-associated region at 1q21.3.

Candidate Gene	Mean melanoma ERC	Empirical P-value
*MCL1*	0.173	0.071
*CERS2*	0.037	0.402
*CTSS*	0.016	0.458
*CTSK*	-0.071	0.695
*ANXA9*	-0.093	0.755
*HORMAD1*	-0.098	0.768
*GOLPH3L*	-0.113	0.800
*ENSA*	-0.187	0.920
*ARNT*	-0.232	0.959
*SETDB1*	-0.280	0.987

### *Evolution-based Disease Map*: ERC signatures reveal genetic relationships between diseases

**Figure 4:**
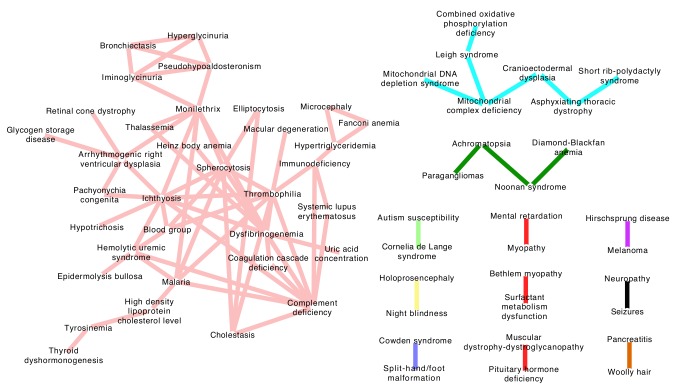
Evolution-based disease map. ERC signatures between diseases were used to draw connections between separate diseases at a false discovery rate of 5%. The 12 clusters represent diseases that involve common genetic mechanisms as inferred by ERC. The largest cluster (pink network) contains several blood-related pathologies, while the light blue network contains mitochondrial diseases and ciliopathies. The remaining clusters contain many novel disease-disease relationships and are addressed fully in the Discussion section.

Having found robust ERC co-evolutionary signatures between genes within a disease, we next sought to draw links between diseases using the same signatures. We hypothesized that such links would cluster diseases with functionally related genes and potentially reveal unforeseen relationships between diseases. Specifically, we inferred a connection between a pair of diseases if the mean ERC value between their constituent genes was significantly elevated compared to random gene sets. To avoid an artificial inflation of the mean ERC value, any genes shared between DGG's were dropped from the calculation. Of the 48,205 disease-disease pairs 132 had significantly elevated ERC at a p-value of 5 × 10^-4^ or lower, which represents a 5.5-fold enrichment. Applying a stringent 5% false discovery rate, there were a total of 81 disease-disease connections, which formed 12 clusters of potentially related diseases (Fig. 4). The resulting “disease map” contained ERC-drawn clusters with strong tendencies to contain diseases with related pathogenic mechanisms. The largest cluster consisted of 34 diseases and could be broadly classified as blood-related disorders (Fig. 4; light red network). The second largest cluster of 7 diseases (light blue) consisted of mitochondrial disorders and ciliopathies, and the third multi-gene cluster was composed of 4 heterogeneous disorders (dark green) that have some shared symptomology relationships. Finally, the 9 remaining clusters were pairs of diseases consisting of a heterogeneous collection of disorders. The significance of these relationships is fully addressed in the Discussion section.

## Discussion

In this study we demonstrate that the relationships between disease-associated genes are often reflected in evolutionary signatures encoded in their gene sequences. Using our metric, evolutionary rate covariation (ERC), and the Online Mendelian Inheritance in Man (OMIM) database, we report 40 diverse diseases whose genes have elevated co-evolutionary signatures at a false discovery rate of 5%, with an additional 130 diseases that also contain elevated rates according to false discovery rate analysis. We found statistically significant elevations of ERC both between genes causing rare Mendelian disorders, such as Fanconi anemia, as well as more common diseases such as Alzheimer's disease, pancreatitis, deafness, colorectal cancer and renal cell carcinoma (Supplemental S1 Table). The signatures we observe likely reflect the close functional relationships between the genes involved in a common pathogenic mechanism. We have observed similar signatures between functionally related genes in diverse biological processes and across different taxonomic groups ranging from single-celled organisms to mammals [26,28,29]. Ultimately, the signatures arise from shared fluctuations in evolutionary rates as the genes respond to changing selective pressures. These observations also suggest that these gene networks have been in tact throughout mammalian evolution and that they evolve together in response to shared evolutionary pressures. Overall, the strong signatures in many diseases led us to test ERC's ability to reveal novel genetic relationships in human diseases.

ERC signatures can be calculated with existing genome sequences and are thus a practical tool to prioritize candidate genes or to infer the function of novel genes. To demonstrate the potential of ERC signatures to prioritize candidate genes for a given disease we again used the OMIM catalog. By treating each OMIM disease gene in turn as a hypothetically unknown disease gene, we examined its mean ERC value with the remaining known genes for its disease, i.e. the training set. Compared to its chromosomal neighbors from a 1-Mb window or to a set of randomly selected genes across the genome, the true disease gene scored higher on average, yet sometimes not high enough to reliably or efficiently prioritize experimental follow-up. However, for diseases with an ERC signature in their training set (p-value < 0.05), the disease gene was prioritized within the top 5 to 15% on average and in many cases was placed in the top position. To assess our prioritization method, we compared our results to a study that analyzed nine prioritization tools that largely rely on text mining, large-scale genomics, proteomics, expression and genetic association datasets [35]. For cases with a significant ERC signal in the training set, ERC performed on par with or exceeded the top methods (Table 3). The fact that ERC uses data that is completely independent of these methods raises the exciting possibility that their integration with ERC would further improve prioritization. There is a notable caveat that success in our method depends on significant ERC within the training set, but fortunately this is a simple calculation that can be performed before any data is gathered, and we estimate that approximately one-quarter of genetic diseases satisfy this requirement (72 of 310 diseases had ERC p-values < 0.05). The potential for ERC to inform and guide experimental efforts in human disease research is mirrored by ERC's previous successes in model organisms [28,31].

**Table 3:** ERC gene prioritization compared to other methods.

Prioritization Tool	Median prioritization rank (%)	% cases in top 30%
**ERC, *P < 0.0001***	**92.6**	**78.7**
**ERC, *P < 0.01***	**82.8**	**64.3**
**ERC, *P < 0.05***	**74.3**	**55.4**
Suspects	87.3	63.0
ToppGene	83.2	52.4
GeneWanderer-RW	77.9	61.9
Posmed-KS	68.5	23.8
GeneDistiller	88.9	78.6
Endeavour-CS	88.8	90.5
Pinta-CS	81.1	71.4

Based on our results here, there are a number of practical guidelines we can prescribe for gene prioritization. Each of these steps can be performed on our public ERC webserver using the 'Gene Prioritization' function, which also provides other ERC-based analysis tools (http://csb.pitt.edu/erc_analysis/). The first step is to define a training set of genes already known to be involved in the disease in question. Notably, chances of success should be improved by predicting likely pathogenic mechanisms when possible from clinical data or cellular phenotypes and choosing the most appropriate genes. The next step is to test for an ERC signature within the chosen training set considering our results showed drastically improved prioritization for diseases with strong signatures—the effect was strong enough that we recommend proceeding only if the training set shows a significantly elevated mean ERC. Based on our survey of OMIM-curated diseases, this requirement should be met by approximately a quarter of diseases with a genetic component. However, a potentially larger proportion of diseases could be interrogated if experts choose discrete pathways with stronger ERC signatures as training sets, possibly through careful examination of molecular phenotypes and integration of other bioinformatics tools. The last step is to calculate the mean ERC value of each candidate with the training set. In our example, this set of steps identified the *MCL1* gene from a melanoma-associated region as the most likely candidate.

Our between-disease analysis of ERC produced a set of disease-disease associations based on evolutionary signatures (Fig. 4). Tight clusters within this disease map reproduced accepted associations between certain diseases; and perhaps more interestingly, ERC associations also uncovered novel evolutionary relationships between clinically distinct diseases. For example, ERC was able to cluster four mitochondrial diseases that were all intuitively related, some being subclasses of the other. Additionally, a triad of clinically related diseases referred to as skeletal ciliopathies—cranioectodermal dysplasia, asphyxiating thoracic dystrophy and short-rib polydactyly syndrome—was found to share significant ERC values not only amongst each other, but ERC also linked these diseases strongly to the mitochondrial disease network [36,37]. The relationship between mitochondrial disorders and ciliopathies is largely unaddressed in the literature, but there are reports that mitochondrial proteins may co-localize with ciliary proteins [38] and there is evidence of a mitochondrial protein deficiency (*XPNPEP3*) that produces a, phenotypically speaking, ciliopathy-like syndrome [39].

Many two-disease clusters also showed compelling, non-intuitive relationships. A link between surfactant metabolism dysfunction and Bethlem myopathy was deemed significant by ERC values, despite these two diseases having very little in common with one another clinically. Bethlem myopathy is caused by a defect in the production of a specialized collagen that leads to debilitating muscle weakness, while inherited surfactant defects leads to severe respiratory deficits. However, recent evidence has interestingly suggested that surfactant proteins have essential collagen domains for surfactant homeostasis [40,41].

A rather dramatic pairing is the association between melanoma and Hirschsprung's disease, an embryologic defect of neural crest cell migration in which a portion of the intestinal nervous system lacks innervation, becomes immotile and causes gastrointestinal obstruction. Again, although these two diseases are clinically distinct, the association of the two using ERC suggests a shared mechanism between them. Digging into this relationship further, strikingly, nearly all genes associated with Hirschsprung's disease have had some evidence in melanoma pathogenesis. Variants in *EDNRB* have been loosely associated with increased melanoma risk in humans and are hypothesized to play a role in CNS melanoma metastases [42,43]. Additionally, if *EDNRB* is heterozygously deleted in a mouse transgenically expressing *RET*—another Hirschsprung disease gene—mice develop *de novo* melanoma lesions [44]. Moreover, yet another Hirschsprung-associated gene, *EDN3*, has also been linked to melanoma invasiveness [45]. Lastly, a research group serendipitously produced a Hirschsprung's disease mouse model while attempting to create a UV-induced melanoma model by knocking out a DNA repair gene in melanocytes of mice, with that same gene now being proposed as a potential mediator of melanoma chemoresistance [46,47]. The relationship between these two diseases is largely unaddressed specifically in the literature, although there is one report of an inherited form of Hirschsprung's disease that had a suspicious pattern of melanoma and pigment abnormalities within the family (Wildin and Eichmeyer, 2008, ASHG, abstract). Melanocytes and enteric nerve cells are known to be both embryologically derived from neural crest cells, perhaps explaining at least in part why there may be an evolutionary link between the shared mechanisms of dysfunction that was uncovered by ERC.

Another connection of interest included one made between Noonan syndrome and Diamond-Blackfan anemia. These two diseases have no obvious pathogenic connection; however, they were linked by ERC. Interestingly, the two share common features including neck webbing, micrognathia, low-set ears, specific cardiac abnormalities and epicanthus among many others [48,49], suggesting ERC may be able to link diseases with shared symptomatology.

The largest cluster consisted of a network of what could be broadly classified as blood-related disorders. With 34 diseases, this group consisted of 63 disease-disease connections. The more intuitive connections included ERC links between inherited disorders that produced erythrocyte structure defects—spherocytosis and eliptocytosis—and also statistically strong links between inherited disorders of hemostasis such as thrombophilia, dysfibrinogenemia and general coagulation cascade deficits. ERC also linked thyroid dyshormogenesis and hypertryosinemia, of note since tyrosine molecules are the synthetic precursors of thyroid hormones. Another particularly interesting connection within this network included a strong link between complement deficiency and systemic lupus erythematosus. Past research has shown a strong link between these two diseases, and here, we show shared evolutionary signatures further corroborating this observation [50,51]. Other intriguing observations can be made, such as a link between atypical uremic syndrome—caused by a loss of inhibitory factors within the complement cascade—and complement protein deficiencies. In summary, these associations imply that ERC can generate large-scale, informative gene-based networks. In this case, we were able to build logical disease networks and uncover potentially novel pathogenic relationships between disease-causing genes using a molecular evolution signature.

Other recent studies have laid out disease associations into maps or networks using different approaches. A pioneering map by Goh *et al*. inferred links between Mendelian diseases based on shared contributing genes, and was able to form an expansive disease network [52]. While our evolution-based map explicitly ignored shared disease genes, it still exhibited a number of disease-disease associations in agreement with the Goh *et al*. map (Supplemental S3 Table). Moreover, our map revealed a number of associations not found in theirs, suggesting that ERC can uniquely uncover linkages between diseases—an example being between melanoma and Hirschsprung's disease as discussed above. Another promising ERC disease map-specific example is a cluster of renal and pulmonary diseases that share solute transport imbalance as a central characteristic—iminoglycinuria, hyperglycinuria, pseudoaldosteronism, and bronchiectasis [53]. A disease map by Suthram *et al*. adopted a sophisticated strategy to discover disease relationships using both protein interaction modules and co-expression profiles [54]. This dual strategy allowed them to move beyond Mendelian diseases and map associations between multi-genic disorders. However, we were unable to compare the evolutionary map with theirs because we examined a different set of diseases. The most recent disease-disease association study departed from genetic data and used massive databases of patient phenotypes to infer relationships between both common diseases and rare Mendelian ones [4]. Most diseases in this map were not found in ours, but of those found in both studies, there was concordance. For example, both maps inferred an interconnected cluster of skin, blood, and immune-related diseases.

Lastly, a future aim of ours is to integrate our approach with other tools currently available. ERC is a unique signature of co-functionality that is entirely derived from comparative sequence analysis. As such, it is expected to be independent and complementary to other established approaches, such as physical interaction datasets, co-expression analyses and literature mining algorithms [5,12,55]. Integrating these methods will allow investigators to capitalize on the strengths of each, enhancing our ability to prioritize and reveal valuable functional information regarding disease genes as well as to further contribute towards the recent trend of network-based studies of genes and diseases [4,56,57].

These efforts broadly begin to demonstrate the profound potential of utilizing a network-based understanding of molecular evolution to assist in gene prioritization, gene functional annotation and informative gene-based network generation. Our hope is that ERC will provide an alternative strategy for biomedical researchers to more efficiently transform gene candidates into actionable hypotheses.

## Materials and Methods

### Mammalian ERC value calculation

ERC values were calculated between 17,486 pairs of human genes as described in previous publications [26,28]. In order to be included in the mammalian ERC analysis, gene ortholog presence was required in a minimum of 17 of the 33 species in the dataset. Of the 19,733 mammalian gene alignments considered, 17,487 met this threshold. Briefly, branch lengths based on amino acid divergence were created from protein coding mammalian sequences derived from the following species: *Homo sapiens* (human), *Pongo pygmaeus abelii* (orang-utan), *Macaca mulatta* (rhesus macaque), *Callithrix jacchus* (marmoset), *Tarsius syrichta* (tarsier), *Microcebus murinus* (mouse lemur), *Otolemur garnettii* (bushbaby), *Tupaia belangeri* (tree shrew), *Cavia porcellus* (guinea pig), *Dipodomys ordii* (kangaroo rat), *Mus musculus* (mouse), *Rattus norvegicus* (rat), *Spermophilus tridecemlineatus* (squirrel), *Oryctolagus cuniculus* (rabbit), *Ochotona princeps* (pika), *Vicugna pacos* (alpaca), *Sorex araneus* (shrew), *Bos taurus* (cow), *Tursiops truncatus* (dolphin), *Pteropus vampyrus* (megabat), *Myotis lucifugus* (microbat), *Erinaceus europaeus* (hedgehog), *Equus caballus* (horse), *Canis lupus familiaris* (dog), *Felis catus* (cat), *Choloepus hoffmanni* (sloth), *Echinops telfairi* (tenrec), *Loxodonta africana* (elephant), *Procavia capensis* (rock hyrax), *Dasypus novemcinctus* (armadillo), *Monodelphis domestica* (opossum), *Macropus eugenii* (wallaby), and *Ornithorhynchus anatinus* (platypus). Branch lengths were estimated using the *aaml* program of the PAML package [58]. These lengths were normalized into relative rates using the projection operator method [59], and correlation coefficients (i.e. ERC values) between these relative rates were calculated between every pair of genes using custom Perl programs.

### OMIM disease gene ERC analysis

Data was downloaded from the OMIM website on June 4, 2013. Using the OMIM Morbid Map dataset, which is a list of diseases followed by single gene associations from published studies, a Perl script was written that grouped disease genes by character matching manually curated disease gene associations into respective disease gene groups. 310 Disease Gene Groupings (DGGs) were generated by broadly grouping all genes with matching disease names, effectively producing a list that consisted of each disease with multiple genes that have been associated with that particular disease. These groups can be found in Supplemental S2 Table, which lists all genes within each DGG along with their corresponding gene and phenotype MIM numbers. From this data, the average ERC value between all combinations of genes within each DGG from the 33 mammalian-species ERC dataset was calculated and then statistically compared to a null distribution of 100,000 random gene groups of the same size using a customized Perl script to determine any significant elevations in the mean ERC value. The analysis was limited to disease gene pairings that were present in the current ERC database (17,487 human genes & 133,416,393 ERC value pairs) and DGGs that contained greater than 1 gene. The data was then sorted by p-value to determine diseases that most significantly harbored elevated ERC signatures. Lastly, a false discovery rate analysis was performed using the 'fdrtool' R package on the resulting p-values [60].

### ERC disease gene prioritization

We assessed ERC's ability to prioritize genes by creating a benchmarking study that generated a list of all genes surrounding a “target” disease gene within a 10 MB region and grouped them into an aggregate “candidate” gene list. Using a “training set” of the remaining OMIM genes shown to be associated with the disease, the candidate genes were then prioritized based on ERC values. We attempted to prioritize the genes using two ERC ranking strategies. The first method (GROUP ERC) calculated the mean ERC value of each candidate gene with all genes in the training set and then ranked the candidates from highest mean ERC to lowest. The second method (BEST ERC) scanned ERC values between each gene in the training set with each candidate gene and used the maximum ERC value between any training set gene to rank the candidates. Ultimately, the GROUP ERC method was chosen for application in the prioritization tests.

### Inferring relationships between diseases with ERC

Disease-disease comparisons were made by calculating the mean ERC value between the genes in each of the two diseases and then comparing that value to that of 10,000 resampled pseudo-disease sets. If two DGG's shared genes, these genes were dropped from the ERC mean calculation to avoid an artificial enhancement of the value. The number of pseudo-datasets greater than or equal to the observed mean were tallied to calculate a permutation p-value. There were a total of 48,205 pairwise comparisons between all 310 Disease Gene Groupings. With the resulting p-values we performed false discovery rate analysis as before [60] and reported all disease-disease pairs significant at a false discovery rate of 5% (Fig. 4).

## Supporting Information

S1 Fig ERC disease gene prioritization—scattered gene distributions. The prioritization of the true disease gene relative to randomly chosen genes throughout the genome improves with a stronger ERC signal within the training set. A low p-value (x-axis) indicates strong ERC within a training set. Prioritization (y-axis) is presented as the proportion of candidate genes scoring lower than the true disease gene, i.e. higher represents better prioritization. The red series is for diseases with training sets with 20 or fewer genes, representing the majority (70%) of OMIM diseases interrogated. The dotted orange line is for those diseases with larger training sets
(TIFF)

S1 Table ERC in all 310 disease gene groupings. Each disease gene grouping (DGG) is presented with the mean ERC between all of its constituent genes. The permutation p-value reflects how likely it is to achieve the indicated mean ERC or higher in random gene sets of the same size. Q-values ('qval') reflect the false discovery rate for that given p-value taking into consideration the full distribution of p-values. 'Ngenes' lists the number of genes in that DGG.
(XLSX)

S2 Table OMIM disease gene groupings. This table provides the Mendelian Inheritance in Man (MIM) numbers for each phenotype and gene associated with a particular Disease Gene Grouping (DGG).
(XLSX)

S3 Table Comparison of disease maps. This supplemental table lists examples of disease-disease associations that were concordant and discordant between the evolution-based (ERC) disease map and the disease map produced by Goh *et al*. (*PNAS* 2007). Each line lists 2 or more diseases that formed an associated cluster. The first list contains disease associations found in both maps. The second contains associations found in our evolution-based map that were not observed in the map by Goh *et al*.
(PDF)
