# Supplementary figures and images for: Evolutionary Signatures amongst Disease Genes Permit Novel Methods for Gene Prioritization and Construction of Informative Gene-Based Networks
Source: PLoS Genet. 2015 Feb 13;11(2):e1004967. doi: 10.1371/journal.pgen.1004967 (PMC4334549; doi:10.1371/journal.pgen.1004967)

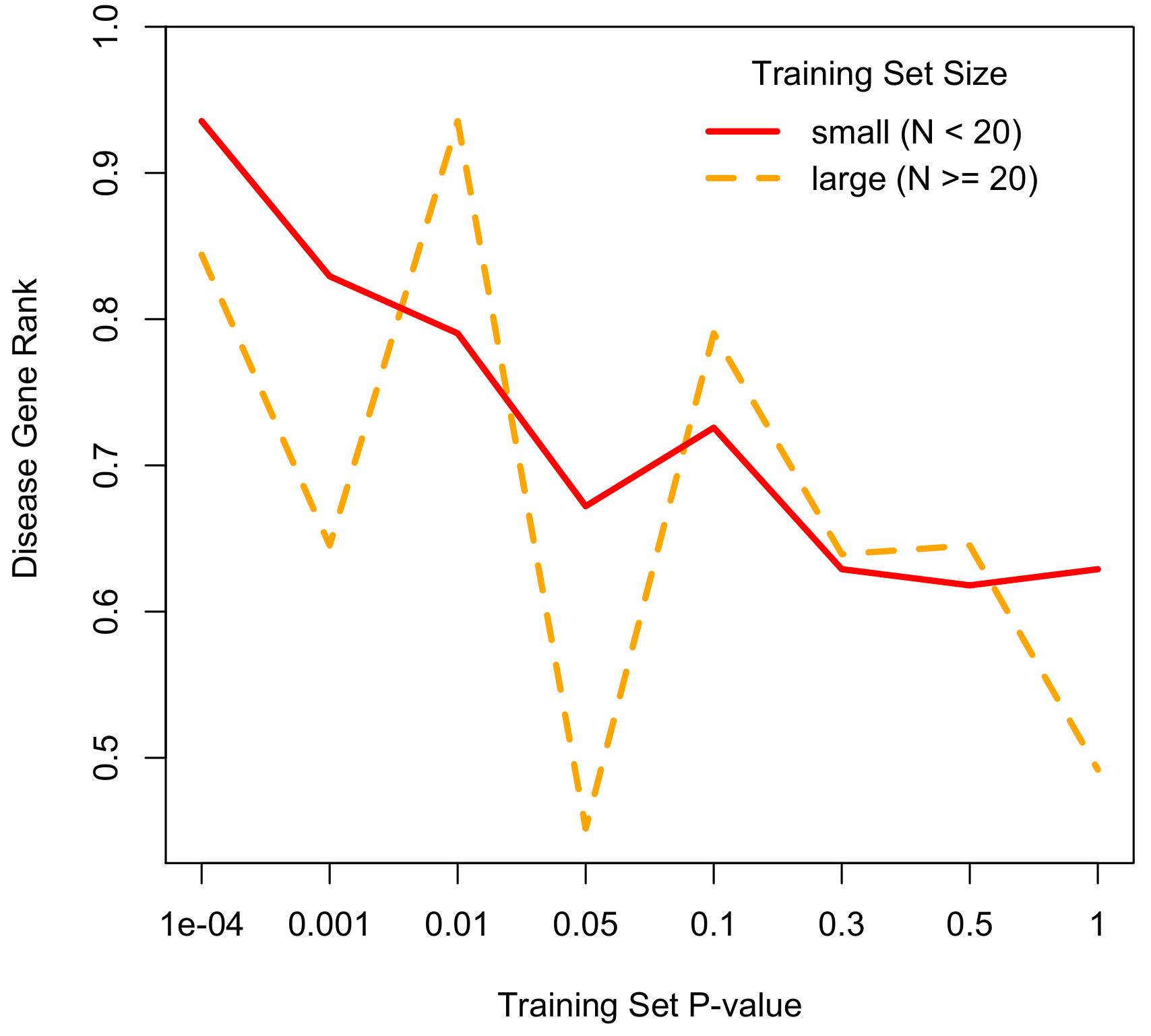

Supplement: S1 Fig — The prioritization of the true disease gene relative to randomly chosen genes throughout the genome improves with a stronger ERC signal within the training set. A low p-value (x-axis) indicates strong ERC within a training set. Prioritization (y-axis) is presented as the proportion of candidate genes scoring lower than the true disease gene, i.e. higher represents better prioritization. The red series is for diseases with training sets with 20 or fewer genes, representing the majority (70%) of OMIM diseases interrogated. The dotted orange line is for those diseases with larger training sets (TIFF) [file pgen.1004967.s001.tiff]
