# Supplementary material for: Evolutionary Signatures amongst Disease Genes Permit Novel Methods for Gene Prioritization and Construction of Informative Gene-Based Networks
Source: PLoS Genet. 2015 Feb 13;11(2):e1004967. doi: 10.1371/journal.pgen.1004967 (PMC4334549; doi:10.1371/journal.pgen.1004967)
Supplement: S3 Table — This supplemental table lists examples of disease-disease associations that were concordant and discordant between the evolution-based (ERC) disease map and the disease map produced by Goh et al. (PNAS 2007). Each line lists 2 or more diseases that formed an associated cluster. The first list contains disease associations found in both maps. The second contains associations found in our evolution-based map that were not observed in the map by Goh et al. (PDF) [file pgen.1004967.s004.pdf]

Table S3 – Comparison of evolutionary and non-evolutionary disease maps

**Disease associations shared between Evolutionary and Goh *et al.* map**
Thalassemia, Heinz body anemia
Thrombophilia, Dysfibrogenemia
Complement deficiency, Immunodeficiency
Holoprosencephaly, Nightblindness (“Ophthamological cluster”)
Blood group, Spherocytosis, Elliptocytosis, Malaria
Ichthyosis, Arrythmogenic right ventricular dysplasia
**Evolutionary disease associations not found in Goh *et al.* map**
Hirschsprung disease, Melanoma
Bethlem myopathy, Surfactant metabolism dysfunction
Mental retardation, Myopathy
Achromatopsia, Paraganglioma
Leigh syndrome, various mitochondrial diseases
Iminoglycinuria, Hyperglycinuria, Pseudoaldosteronism, Bronchiectasis
